# LncRNA BANCR promotes tumorigenesis and enhances adriamycin resistance in colorectal cancer

**DOI:** 10.18632/aging.101530

**Published:** 2018-08-22

**Authors:** Siping Ma, Dongxiang Yang, Yanlong Liu, Yongpeng Wang, Tao Lin, Yanxi Li, Shihua Yang, Wanchuan Zhang, Rui Zhang

**Affiliations:** 1 Department of Colorectal Surgery, Cancer Hospital of China Medical University, Liaoning Cancer Hospital & Institute, Shenyang 110042, China; 2 Department of Orthopedics, The Affiliated Hospital of Liaoning University of Traditional Chinese Medicine, Shenyang 110032, China; 3 Department of Colorectal Surgery, Harbin Medical University Cancer Hospital, Harbin 150081, China

**Keywords:** long non-coding RNA, colorectal cancer, BANCR, microRNA-203, CSE1L, adriamycin, chemoresistance

## Abstract

Colorectal cancer (CRC) is the third most common malignancy in the United States. Chemotherapeutic resistance is a massive obstacle for cancer treatment. The roles and molecular basis of long non-coding RNA BRAF-activated noncoding RNA (BANCR) in CRC progression and adriamycin (ADR) resistance have not been extensively identified. In this study, we found that BANCR and CSE1L expressions were upregulated in CRC tumor tissues. Meanwhile, CSE1L expression was correlated with depth of CRC. BANCR silencing suppressed cell proliferation and invasion capacity, increased apoptotic rate and potentiated cell sensitivity to ADR. CSE1L downregulation triggered a reduction of cell proliferation and invasion ability, and an increase of apoptosis rate and cell sensitivity to ADR. CSE1L overexpression attenuated si-BANCR-mediated anti-proliferation, anti-invasion and pro-apoptosis effects in CRC cells. BANCR acted as a molecular sponge of miR-203 to sequester miR-203 away from CSE1L in CRC cells, resulting in the upregulation of CSE1L expression. CSE1L knockdown inhibited expressions of DNA-repair-related proteins (53BP1 and FEN1) in HCT116 cells. BANCR knockdown also inhibited tumor growth and enhanced ADR sensitivity in CRC mice model. In conclusion, BANCR knockdown suppressed CRC progression and strengthened chemosensitization of CRC cells to ADR possibly by regulating miR-203/CSE1L axis, indicating that BANCR might be a promising target for CRC treatment.

## Introduction

Colorectal cancer (CRC) is the third most common malignancy and the third leading cause in cancer-induced deaths in the United States with an estimated 135,430 new cases and 50,260 deaths in 2017 [1,2]. Both genetic and environmental changes have been considered to be involved in the etiology of CRC [3].

Despite substantial advances in treatment options, such as surgery, radiotherapy and chemotherapy, the cure rates and long-term survival of CRC remain unsatisfactory [4]. Therefore, it is still imperative to identify more effective biomarker and molecular target for improving CRC therapy.

Long non-coding RNAs (lncRNAs), a class of transcripts longer than 200 nucleotides without protein-coding potential, have been identified as critical mediators in the development and progression of cancers [5]. The dysregulation of lncRNAs has been highlighted to be implicated in a serial of cellular processes and signaling pathways associated with CRC etiopathogenesis [6]. BRAF-activated noncoding RNA (BANCR), a 693 bp lncRNA located on chromosome 9, has been identified as an oncogene or a tumor suppressor in a variety of human malignancies, such as lung cancer, gastric cancer, colorectal cancer, thyroid cancer, melanoma, hepatocellular carcinoma and osteosarcoma [7]. The roles of BANCR in CRC are controversial in previous literatures. For example, some researchers pointed out that BANCR was highly expressed in CRC tissues and cell lines, and BANCR overexpression induced cell migration by facilitating the transition of epithelial to mesenchymal (EMT) via an ERK-dependent mechanism [8]. Moreover, BANCR was found to be up-regulated in CRC tissues, and associated with lymph node metastasis and poor survival of CRC patients [9]. On the contrary, Shi et al. demonstrated that BANCR level was strikingly decreased in CRC tissues and cell lines, and ectopic expression of BANCR suppressed cell proliferation and tumor xenograft growth, and induced cell cycle arrest and apoptosis by increasing p21 expression in CRC [10]. Hence, in the present study, we aimed to further investigate the roles and molecular basis of BANCR in CRC progression.

Human chromosomal segregation 1-like (CSE1L) gene, also named as human cellular apoptosis susceptibility (CAS) or exportin-2 gene, maps on chromosome 20q13 [11]. CSE1L, highly expressed in various cancer types, plays important roles in apoptosis, cell survival, chromosome assembly, nucleocytoplasmic transport, microvesicle formation, and cancer metastasis [12,13]. In CRC, CSE1L expression was upregulated and CSE1L knockdown suppressed cell proliferation, metastasis, and induced apoptosis [14–16]. However, whether the effect of CSE1L on CRC pathogenesis was mediated by BANCR is still obscure.

In this study, we firstly demonstrated that BANCR and CSE1L expressions were both up-regulated in CRC tumor tissues, and CSE1L expression was positively associated with BANCR expression and clinicopathological factors of CRC. Consequently, the roles and molecular mechanisms of BANCR in CRC cell proliferation, invasion, apoptosis and chemoresistance were further explored.

## RESULTS

### BANCR and CSE1L expressions were upregulated in CRC tumor tissues

**Figure 1:**
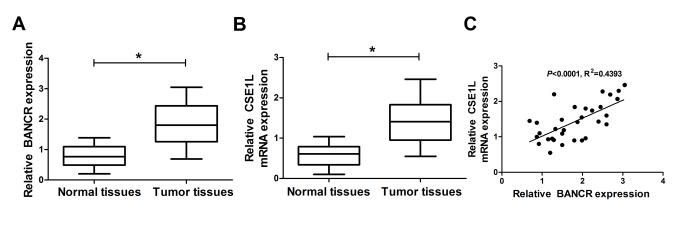
**BANCR and CSE1L were highly expressed in CRC tumor tissues.** (**A** and **B**) Expressions of BANCR and CSE1L in 32 pairs of CRC tumor tissues and adjacent normal tissues. (**C**) Correlation analyses of BANCR and CSE1L expressions in CRC tumor tissues (n=21). *P < 0.05.

Firstly, RT-qPCR assay was performed to measure expression patterns of BANCR and CSE1L in 32 pairs of CRC tumor tissues and adjacent normal tissues. Results showed that BANCR and CSE1L expressions were both significantly upregulated in CRC tumor tissues (n=32) compared with adjacent normal tissues (n=32) (Fig. 1A and 1B). However, little change of pCSE1L/CSE1L ratio was observed between CRC tumor tissues and normal group, suggesting that phosphorylated CSE1L may not be involved in CRC development (Suppl. Fig. 1A). Moreover, CSE1L expression was positively associated with BANCR expression in 32 cases of CRC tumor tissues (Fig. 1C). To probe the association of CSE1L expression with clinicopathologic features, the 32 patients with CRC were then classified in Table 1. Result showed that CSE1L expression was associated with depth of tumor (p<0.05). Nevertheless, the expression of CSE1L was independent of age, gender, size, stages or location (p>0.05). These results hinted that BANCR and CSE1L might participate in the regulation of CRC progression.

**Table 1:** Association of CSE1L expression with clinicopathological factors in colorectal cancer.

Clinicopathological feature	Number	Relative expression of CSE1L	*p* value
**Age (years)**			
≤ 60	18	1.38±0.52	0.5694
> 60	14	1.49±0.54	
**Gender**			
Female	13	1.43±0.50	0.6292
Male	19	1.52±0.54	
**size (cm)**			
> 5	12	1.47±0.49	0.7094
≤ 5	20	1.40±0.55	
**stage**			
I	2	1.23±0.59	0.3380
II	12	1.42±0.65	
III	14	1.39±0.46	
IV	4	1.68±0.38	
**location**			
colon	14	1.38±0.50	0.8919
rectum	18	1.35±0.54	
**depth**			
T1/T2	22	1.27±0.50	0.0093*
T3/T4	10	1.77±0.41	

Notes: Relative expression of CSE1L was calculated using 2^−∆∆Ct^ method. Data were shown as mean ± standard deviation, *p < 0.05.

### BANCR knockdown suppressed proliferation and invasion, induced apoptosis, and potentiated chemosensitivity in CRC cells

**Figure 2:**
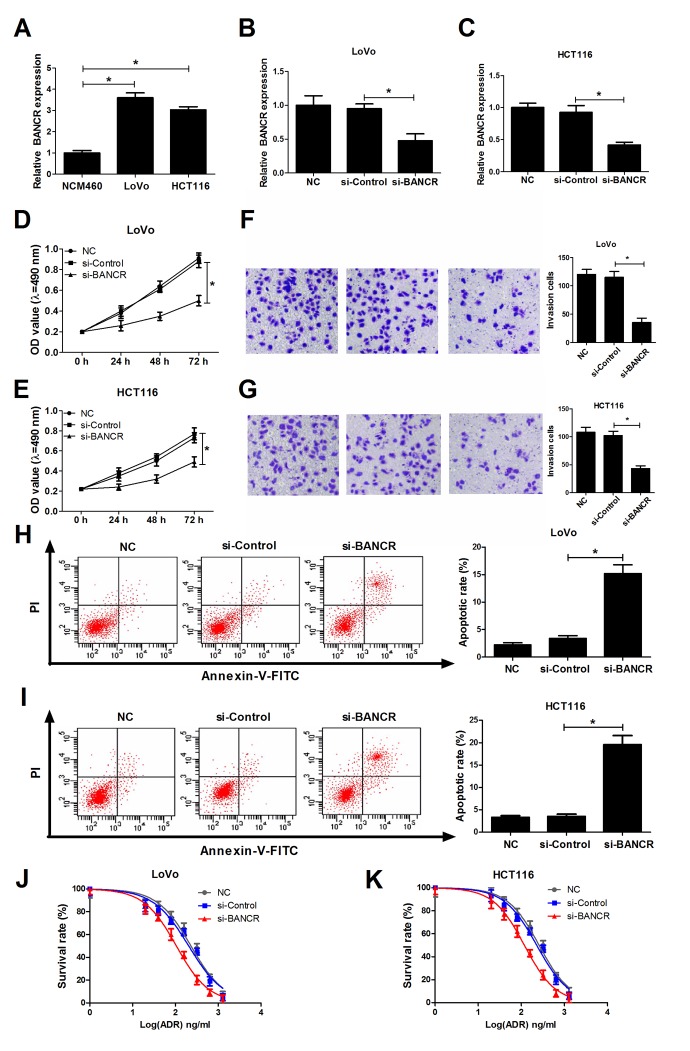
**BANCR knockdown suppressed invasion, proliferation, induced apoptosis and increased ADR sensitivity in CRC cells.** (**A**) Expression of BANCR in human normal colon mucosal epithelial cell line (NCM460) and CRC cell lines (LoVo and HCT116) was detected using RT-qPCR assay. (B-K) LoVo and HCT116 cells were transfected with si-Control or si-BANCR with untransfected (NC) or si-Control-transfected cells acted as blank or mock control, respectively. (**B** and **C**) Knockdown efficiency of si-BANCR was evaluated by RT-qPCR assays at 48 h upon transfection. (**D** and **E**) The effect of BANCR depletion on proliferation ability was measured by MTT assay at the indicated time points (0, 24, 48, 72 h) upon transfection in LoVo and HCT116 cells. (**F** and **G**) The effect of BANCR knockdown on invasion capability was assessed at 48 h after transfection by transwell invasion assay in LoVo and HCT116 cells. (**H** and **I**) The effect of BANCR deficiency on apoptotic rate was detected in LoVo and HCT116 cells at 48 h posttransfection by flow cytometry via double-staining of Annexin-V-FITC and PI. (**J** and **K**) LoVo and HCT116 cells were treated with different concentrations of ADR (0, 20, 40, 80, 160, 320, 640, 1280 ng/ml) for 48 h, followed by the determination of cell survival rate using MTT assay. *P < 0.05.

Then, we further demonstrated that BANCR expression was significantly increased in CRC cell lines (LoVo and HCT116) compared to that in human normal colonic epithelial cell line (NCM460) (Fig. 2A). To further explore the roles of BANCR in CRC development, si-RNA targeting BANCR (si-BANCR) and its scramble control (si-Control) were synthesized and transfected into LoVo and HCT116 cells, followed by the detection of knockdown efficiency. Results disclosed that BANCR expression was notably decreased in si-BANCR-transfected LoVo and HCT116 cells in comparison with that in untransfected (NC) or si-Control-transfected (mock) cells (Fig. 2B and 2C). Subsequently, we further explored the effects of BANCR down-regulation on biological behavior in CRC cells. MTT assay manifested that knockdown of BANCR markedly inhibited proliferation ability of LoVo and HCT116 cells when compared to control groups (Fig. 2D and 2E). Matrigel invasion assay revealed that the invasive capability was notably reduced in BANCR-silenced LoVo and HCT116 cells compared to that in untransfected or mock cells (Fig. 2F and 2G). Moreover, introduction of si-BANCR led to a significant increase of apoptosis rate in LoVo and HCT116 cells (Fig. 2H and 2I). LncRNAs have been elucidated to affect the occurrence and development of cancer drug resistance properties via modulating multiple targets and pathways [17,18]. Therefore, the effects of BANCR depletion on sensitivity of LoVo and HCT116 cells to ADR were explored by MTT assays. Resulted showed that ADR suppressed cell viability in a dose-dependent manner at the concentration ranging from 0 ng/ml to 1280 ng/ml in LoVo and HCT116 cells. Moreover, depletion of BANCR enhanced sensitivity of LoVo and HCT116 cells to ADR, revealed by the decrease of cell survival rate in BANCR-silenced cells (Fig.2J and 2K). In a word, these results suggested that down-regulation of BANCR inhibited proliferation and invasion, facilitated apoptosis and increased ADR sensitivity in CRC cells.

### CSE1L down-regulation resulted in a reduction of invasion and proliferation capacities and an increase of apoptosis and chemosensitivity in CRC cells

**Figure 3:**
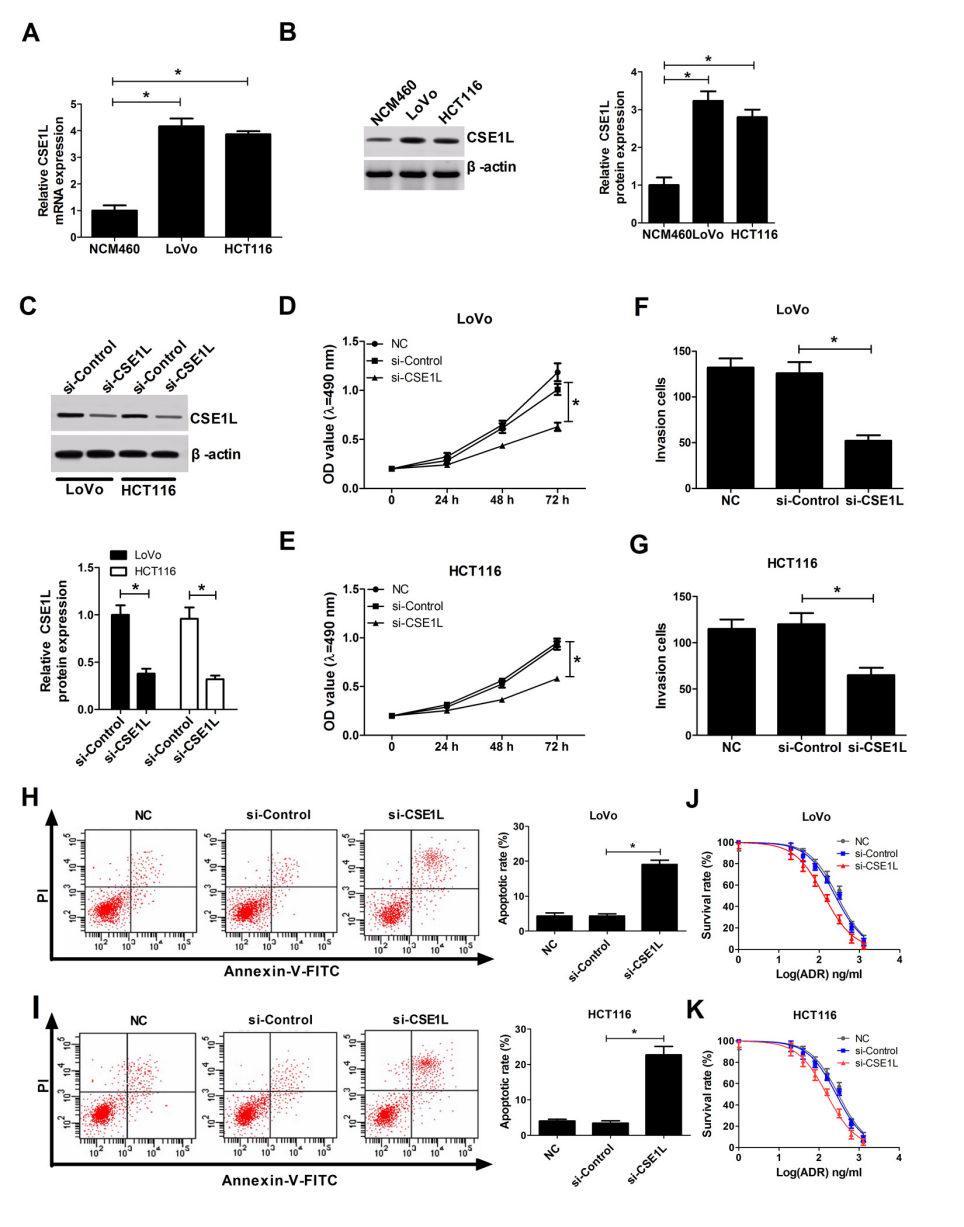
**CSE1L downregulation resulted in a reduction of invasion and proliferation capacities, and an increase of apoptosis rate and ADR sensitivity in CRC cells.** (**A** and **B**) CSE1L expressions at mRNA and protein levels were measured by RT-qPCR and western blot assays in NCM460, LoVo and HCT116 cells. (**C**) LoVo and HCT116 cells were transfected with si-Control or si-CSE1L, followed by measurement of CSE1L protein level via western blot assay at 48 h upon transfection. (**D** and **E**) The effect of CSE1L silencing on proliferation was assessed by MTT analysis in LoVo and HCT116 cells. (**F** and **G**) The effect of CSE1L knockdown on invasion was detected via transwell invasion assay in LoVo and HCT116 cells. (**H** and **I**) The effect of CSE1L deficiency on apoptotic rate was tested by flow cytometry in LoVo and HCT116 cells. (**J** and **K**) LoVo and HCT116 cells were transfected with si-Control or si-CSE1L for 24 h, then untransfected or transfected cells were treated with different concentrations of ADR (0, 20, 40, 80, 160, 320, 640 and 1280 ng/ml) for another 48 h, followed by the detection of cell survival rate using MTT assay. *P < 0.05.

As we might expect, CSE1L expressions at mRNA and protein levels were up-regulated in LoVo and HCT116 cells relative to NCM460 cells (Fig. 3A and 3B). To further inquire the functions of CSE1L in CRC, siRNA of CSE1L (si-CSE1L) was synthesized and introduced into LoVo and HCT116 cells, followed by the measurement of transfection efficiency. Results manifested that introduction of si-CSE1L induced a marked decrease of CSE1L expression in LoVo and HCT116 cells (Fig. 3C), suggesting that si-CSE1L could be employed for the subsequent loss-of-function assays. Functional assays revealed that CSE1L knockdown prominently hindered proliferation (Fig. 3D and 3E), suppressed invasion (Fig. 3F and 3G) and facilitated apoptosis (Fig. 3H and 3I) in LoVo and HCT116 cells. Next, we further proved that depletion of CSE1L increased sensitivity of LoVo and HCT116 cells to ADR, presented by the decline of cell survival rate in si-CSE1L-transfected cells with various concentrations of ADX treatment (Fig. 3J and 3K). Taken together, these data indicated that CSE1L knockdown repressed proliferation and invasion, promoted apoptosis and conferred sensitivity to ADR in CRC cells.

### CSE1L overexpression abrogated si-BANCR-mediated anti-proliferation, anti-invasion and pro-apoptosis effects in CRC cells

**Figure 4:**
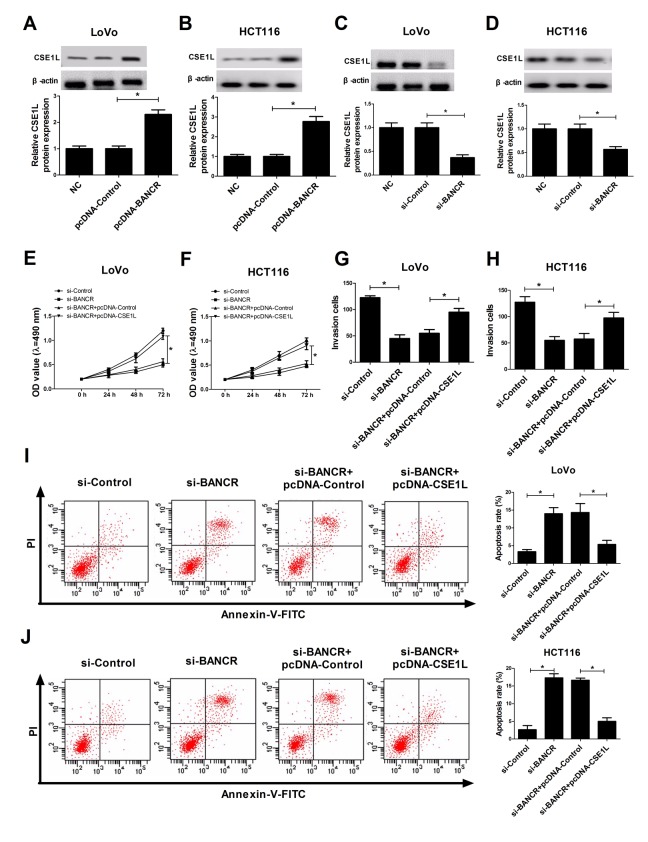
**CSE1L overexpression abrogated si-BANCR-mediated anti-invasion, anti-proliferation and pro-apoptosis effects in CRC cells.** (**A** and **B**) The effect of BANCR overexpression on CSE1 L protein level was detected in LoVo and HCT116 cells. (**C** and **D**) The effect of BANCR knockdown on CSE1 L protein expression was measured in LoVo and HCT116 cells. (**E**-**J**) LoVo and HCT116 cells were transfected with si-Control, si-BANCR, si-BANCR+pcDNA-Control, si-BANCR+pcDNA-CSE1L, followed by the determination of cell viability (**E** and **F**), invasion capacity (**G** and **H**) and apoptosis rate (I and J). *P < 0.05.

Then, we further analyzed the effects of BANCR on CSE1L expression in CRC cells. The results stated that ectopic expression of BANCR triggered a dramatic elevation of CSE1L level in LoVo and HCT116 cells compared to that in untransfected or pcDNA-Control-transfected cells (Fig. 4A and 4B). On the contrary, siRNA-mediated BANCR silencing induced a notable reduction of CSE1L expression in LoVo and HCT116 cells in comparison with that in untransfected or si-Control-transfected cells (Fig. 4C and 4D). Additionally, the phosphorylated CSE1L level was also detected in HCT116 and LoVo cells transfected with BANCR-overexpression plasmids. The result showed that BANCR enrichment displayed little effect on ratio of pCSE1L/CSE1L (Suppl. Fig. 1B and 1C). Moreover, restoration experiments clarified that CSE1L overexpression markedly reversed BANCR-downregulation-induced anti-proliferation (Fig. 4E and 4F), anti-invasion (Fig. 4G and 4H) and pro-apoptosis (Fig. 4I and 4J) effects in LoVo and HCT116 cells, demonstrated by enhanced cell viability (Fig. 4E and 4F), increased invasion number (Fig. 4G and 4H) and reduced apoptosis rate (Fig. 4I and 4J) in BANCR-depleted CRC cells following CSE1L up-regulation. In a word, these data indicated that BANCR affected proliferation, invasion and apoptosis of CRC cells partly through regulating CSE1L.

### BANCR acted as a molecular sponge of miR-203 to sequester miR-203 away from CSE1L in CRC cells

Next, to further explore molecular mechanisms of BANCR affected CSE1L in CRC progression, an online predicted website (https://genie.weizmann.ac.il/pubs/mir07/index.html) was used to search for miRNAs possessing a potential to interact with BANCR. Among candidate miRNAs, miR-203 (Fig. 5A) was selected because that miR-203 performed as a tumor suppressor in multiple cancers including CRC [19,20]. To further validate the potential interaction of BANCR and miR-203, wide-type BANCR reporter (WT-BANCR) containing predicted miR-203 binding sites and mutant-type BANCR reporter (Mu-BANCR) containing mutant miR-203 binding sites were constructed. Subsequent luciferase assays revealed that miR-203 overexpression significantly decreased luciferase activity of WT-BANCR reporter, but had no effect on luciferase activity of Mu-BANCR reporter in LoVo and HCT116 cells (Fig. 5B and 5C), suggesting that BANCR could interact with miR-203 by putative binding sites in CRC cells. It is widely accepted that miRNAs can exert their roles by regulating target gene expressions. Predicted results by TargetScan online website manifested that there existed some complementary sites between miR-203 and CSE1L 3’UTR (Fig. 5D). To further verify the prediction, CSE1L 3’UTR-WT and CSE1L 3’UTR-Mu reporters were generated. Luciferase assays discovered that luciferase activity of CSE1L 3’UTR-WT reporter was significantly suppressed in miR-203-overexpressed LoVo and HCT116 cells, however, these effects were disappeared when the binding sites of miR-203 within CSE1L 3’UTR were mutated. (Fig. 5E and 5F), indicating that CSE1L was a target of miR-203 in CRC cells. Mounting evidence highlights that lncRNAs function as molecular sponges of miRNAs to exert their regulatory effect on target mRNAs [21]. Hence, we further demonstrated the mutual effects of BANCR, miR-203 and CSE1L. qRT-PCR analysis disclosed that miR-203 expression was markedly increased in BANCR-silenced LoVo and HCT116 cells, but was notably decreased in BANCR-overexpressed cells (Fig. 5G and 5H). Moreover, western blot results confirmed that miR-203 up-regulation suppressed CSE1L expression, while miR-203-mediated inhibitory effect on CSE1L expression was reversed by BANCR overexpression in LoVo and HCT116 cells (Fig. 5I and 5J), indicating that BANCR acted as a miR-203 sponge to sequester miR-203 away from CSE1L in CRC cells. Moreover, miR-203 expression was substantially decreased in CRC tumor tissues compared with adjacent non-cancerous tissues (Fig. 5K). Additionally, Spearman’s test found that there existed a negative correlation between miR-203 and BANCR (Fig. 5L) or CSE1L (Fig. 5M) in CRC tumor tissues. Previous studies indicated that CSE1L was related with DNA repair [22,23]. Consequently, the effect of CSE1 knockdown on expressions of DNA repair-related proteins (53BP1 and FEN1) was investigated in HCT116 cells. Results showed that FEN1 and 53BP1 expressions were down-regulated in HCT116 cells following inhibition of CSE1 expression, implicating that CSE1L knockdown prevented the repair of DNA damage.

**Figure 5A-F:**
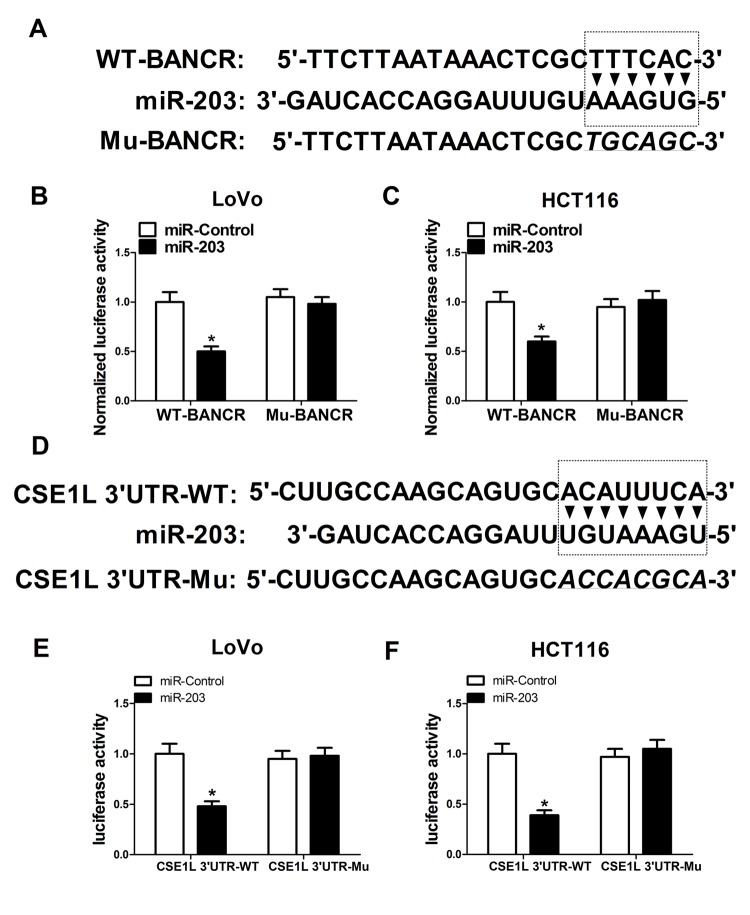
**BANCR acted as a molecular sponge of miR-203 to sequester miR-203 away from CSE1L in CRC cells.** (**A**) Predicted binding sites between BANCR and miR-203, and mutant sites in Mu-BANCR reporter. (**B** and **C**) The effects of miR-203 overexpression on luciferase activity of WT-BANCR and Mu-BANCR reporters were detected in LoVo and HCT116 cells. (**D**) Putative binding sequences between miR-203 and CSE1L 3’UTR, and mutant sites in CSE1L 3’UTR-Mu reporter. (**E** and **F**) The effects of miR-203 overexpression on luciferase activity of CSE1L 3’UTR-WT and CSE1L 3’UTR-Mu reporters were determination in LoVo and HCT116 cells.

**Figure 5G-N:**
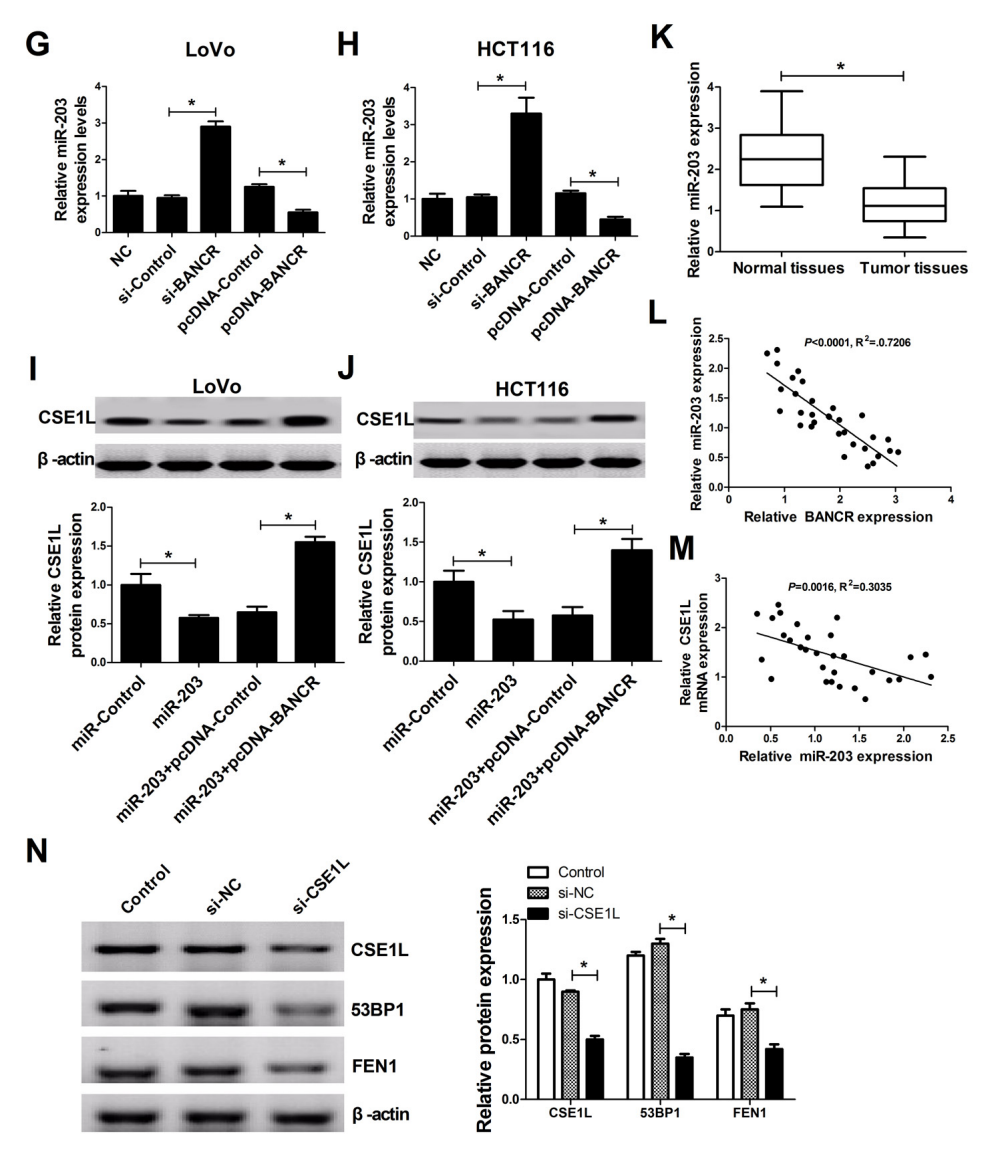
**BANCR acted as a molecular sponge of miR-203 to sequester miR-203 away from CSE1L in CRC cells.** (**G** and **H**) The effects of BANCR knockdown and overexpression on miR-203 expression were assessed in LoVo and HCT116 cells. (**I** and **J**) LoVo and HCT116 cells were transfected with miR-Control, miR-203, miR-203+pcDNA-Control, or miR-203+pcDNA-BANCR, followed by the measurement of CSE1L protein level. (**K**) miR-203 expression in 32 paired CRC tumor tissues and adjacent non-cancerous tissues. (**L** and **M**) Correlation analysis between miR-203 and BANCR or CSE1L in 32 paired CRC tumor tissues. (**N**) The effects of CSE1L silencing on 53BP1 and FEN1 expressions were tested in HCT116 cells. *P < 0.05.

### BANCR knockdown inhibited tumor growth and enhanced ADR sensitivity in CRC *in vivo*

**Figure 6:**
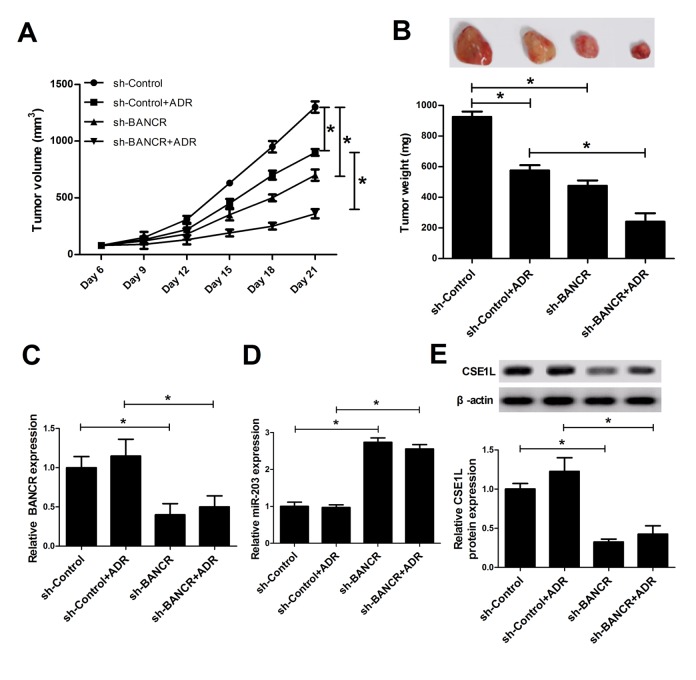
**BANCR knockdown inhibited tumor growth and enhanced ADR sensitivity by modulation of miR-203/ CSE1L pathway *in vivo*.** LoVo/DOX cells stably infected lenti-sh-XIST or lenti-sh-Control were subcutaneously inoculated into, followed by injection with the left flank of mice, ADR (1 mg/kg) every 3 days on day 6 after cell inoculation. (**A**) Tumor volume was determined at the indicated time points (6, 9, 12, 15, 18, 21 days) after first injection. (**B**) At 21 days upon cell implantation, tumors were excised, imaged and weighted. (**C**-**E**) Expressions of BANCR, miR-203 and CSE1L in xenograft tumors were determined by RT-qPCR and western blot assays. *P < 0.05.

Next, mice xenograft models of CRC were established to explore the influence of BANCR knockdown on tumor growth and ADR sensitivity. Results showed that ADR injection or BANCR knockdown curbed tumor growth, and BANCR silencing enhanced ADR-induced anti-tumor effect *in vivo* (Fig. 6A and 6B). Moreover, we further demonstrated that BANCR and CSE1L expressions were decreased (Fig. 6C and 6E), while miR-203 expression was increased (Fig. 6D) in tumor tissues derived from sh-BANCR-transfected HCT116 cells. That is to say, BANCR knockdown inhibited tumor growth and induced ADR sensitivity in CRC possibly by modulating miR-203/CSE1L axis.

## DISCUSSION

CRC accounts for approximately 8% in all new cancer cases and cancer-related deaths in the United States in 2016 [2]. With the improvements of screening, diagnosis, early detection and treatment, a significant reduction of incidence and mortality of CRC has been obtained [24]. However, the prognosis of CRC patients was poor by virtue of the late presentation and chemotherapeutic resistance [25]. Thus, it is of great significance to seek more efficient intervention strategies for CRC.

Accumulative evidence has manifested that lncRNAs are essential regulators of oncogenesis in CRC, offering a possibility of lncRNAs as biomarkers for diagnosis, prognosis and therapy of CRC [26,27]. Previous studies revealed the dysregulation of BANCR in various cancers [28]. For instance, Li et al. found that BANCR was overexpressed in melanoma cell lines and tissues, and BANCR knockdown suppressed melanoma cell proliferation *in vitro* and hindered xenograft tumor growth *in vivo* [29]. Sun et al. demonstrated that BANCR expression was downregulated in non-small cell lung cancer (NSCLC) tissues and cell lines, and BANCR overexpression induced a inhibition of cell proliferation, migration and invasion, a increase of apoptosis rate, and a decline of tumor xenograft growth and metastasis in NSCLC [30].

Previous documents showed that CSE1L was implicated in tumorigenesis by acting as an oncogenic gene in some cancers. For example, CSE1L silencing inhibited osteosarcoma cell proliferation *in vitro* and hampered tumor growth in osteosarcoma xenograft models [31]. Also, CSE1L expression was increased in ovarian cancer, and the depletion of CSE1L impaired invasion and metastasis of ovarian cancer cells [32]. Moreover, as mentioned above, CSE1L was reported to be closely linked with the occurrence and progression CRC [14–16].

In the present study, we firstly demonstrated that BANCR and CSE1L expressions were both upregulated in CRC tumor tissues and cells, consistent with earlier studies [8,9,14–16]. Moreover, CSE1L level was positively associated with BANCR level in CRC tumor tissues. Besides, we found CSE1L expression was correlated with depth of tumor in CRC, consistent with former work [33]. However, they also described a correlation of CSE1L expression with cancer stages. We hypothesized the discrepancy may result from fewer patient cases and larger error in stage I. Functional analyses revealed that siRNA-mediated BANCR silencing resulted in the decrease of proliferation and invasion potency and the increase of apoptosis rate in CRC cells. Additionally, results also showed that BANCR knockdown potentiated ADR sensitivity in LoVo and HCT116 cells. In other words, BANCR performed as an oncogene in CRC, in accordance with some previous studies [8,9]. However, another research elucidated a contradictory result showing that BANCR expression was lowered in colorectal cancer tissues, and enforced expression of BANCR repressed colon cancer cell growth *in vitro* and *in vivo*, indicating its tumor-suppressing property [10]. These controversial conclusions may be attributed to different sample size or microsatellite instability statuses of tumor tissues.

Next, we further substantiated that CSE1L knockdown suppressed proliferation, invasion and induced apoptosis in CRC cells, in agreement with some previous reports [14–16]. Moreover, CSE1L knockdown enhanced sensitivity of LoVo and HCT116 cells to ADR, contradictory with earlier studies pointing out that CSE1L could enhance ADR-induced apoptotic effect in HT-29 cells [34,35]. The results might be associated with the differential expression of CSE1L in different CRC cells. For instance, CSE1L expression was upregulated in HCT116 cells, but was downregulated in HT-29 cells [16]. Moreover, we found that upregulation of BANCR level promoted CSE1L expression, whereas BANCR depletion suppressed CSE1L expression in CRC cells. A previous research demonstrated that phosphorylated CSE1L was associated with malignant melanoma progression [36]. However, we found that pCSE1L/CSE1L ratio had little change in CRC tissues and cells, indicating that phosphorylated CSE1L might not be a key driver in CRC tumorigenesis. In agreement our data, it was found that non-phosphorylated CSE1L played an important role in regulating cell viability and apoptosis in CRC [14]. Furthermore, restoration of CSE1L expression abated si-BANCR-mediated anti-proliferation, anti-invasion, and pro-apoptosis effect in CRC cells.

It is widely accepted that lncRNAs can act as ceRNAs of miRNAs to sequester miRNAs from target mRNAs, resulting in the upregulation of mRNA levels. Hence, online prediction website (https://genie.weizmann.ac.il/pubs/mir07/index.html) was used to search for miRNAs possessing a potential to interact with BANCR. Among candidate miRNAs, miR-203 was selected due to its tumor-suppressing role in multiple cancers such as esophageal squamous cell carcinoma (ESCC) [37], prostate cancer [38] and laryngeal squamous cell cancer [39]. Moreover, miR-203 expression was strikingly downregulated in CRC tissues and cell lines [40,41], and ectopic expression of miR-203 suppressed proliferation, migration and invasion, while promoted apoptosis in CRC cells [19,20]. However, Ju et al. discovered that serum level of miR-203 was increased, and high miR-203 expression indicated a poor prognosis and an advanced status in CRC [42]. Additionally, miR-203 was closely linked with drug resistance in CRC. For instance, miR-203 was highly expressed in oxaliplatin-resistant CRC cell lines, and miR-203 overexpression induced oxaliplatin resistance by targeting ataxia telangiectasia mutated (ATM) kinase in CRC cells [43]. On the contrary, Li et al. disclosed that miR-203 expression was reduced in 5-fluorouracil (5-FU)-resistant cell line and miR-203 downregulation strengthened 5-FU resistance by targeting thymidylate synthase (TYMS) in CRC cells [44]. Additionally, CSE1L-3’UTR was predicted to harbor a binding sites of miR-203. The following luciferase assays showed that enforced expression of miR-203 induced a decrease in luciferase activity of WT-BANCR and CSE1L 3’UTR-WT reporters, but had no effect on luciferase activity of Mu-BANCR and CSE1L 3’UTR-Mu reporters in CRC cells. Additionally, we further confirmed that BANCR knockdown promoted miR-203 expression, while BANCR overexpression suppressed miR-203 expression in CRC cells. Furthermore, miR-203 inhibited CSE1L expression in CRC cells, while this effect was reversed by BANCR upregulation. In other words, these data indicated that BANCR could perform as a molecular sponge of miR-203, resulting in the reduction of miR-203 expression and the increase of CSE1L expression in CRC cells. Moreover, miR-203 expression was decreased in CRC tumor tissues when compared to adjacent non-cancerous tissues. Additionally, an inverse correlation was observed between miR-203 and BANCR or CSE1L in CRC tumor tissues.

Previous studies revealed that CSE1L knockdown inhibited transcription of p53 target genes including p53 inducible gene 3 (PIG3) [22], and PIG3 silencing increased sensitivity of cells to DNA damage agents and impaired DNA repair [23]. Moreover, PIG3 facilitated the recruitment of 53BP1 [23], which contributed to DNA repair [45]. Hence, we further explored whether CSE1L was involved in mediating DNA repair response in CRC. Results showed that FEN1 and 53BP1 expressions were downregulated in CSE1L-depleted RCC cells, indicating that CSE1L knockdown suppressed the repair of DNA damage.

Next, *in vivo* assays further revealed that ADR injection or BANCR knockdown curbed tumor growth, and BANCR silencing could enhance ADR-induced anti-tumor effect in mouse xenograft model of CRC. Moreover, BANCR depletion triggered an elevation of miR-203 expression and a reduction of CSE1L level, suggesting that BANCR downregulation repressed tumor growth and enhanced ADR sensitivity via regulation of miR-203/CSE1L pathway *in vivo.*

In conclusion, our study elucidated that BANCR silencing hampered CRC progression and enhanced ADR sensitivity at least partly by regulating miR-203/CSE1L *in vitro* and *in vivo.* Targeting BANCR might be a potential therapeutic target for CRC management.

## MATERIALS AND METHODS

### Clinical specimens and cell culture

Thirty-two pairs of CRC tumor tissues and adjacent normal tissues were collected from CRC patients without any treatment prior to surgery at Liaoning Cancer Hospital & Institute from 2014 to 2016. Our study was performed with the approval of Ethics Committee of Liaoning Cancer Hospital & Institute and the signed consent from each participant. Resected tumor specimens were snap frozen in liquid nitrogen and then preserved at -80°C. The detailed clinical characteristics of patients are described in Table 1.

Human CRC cell lines (HCT116 and LoVo) were purchased from China Center for Type Culture Collection (CCTCC, Wuhan, China). Human normal colon mucosal epithelial cell line NCM460 was obtained from INCELL Corporation (San Antonio, TX, USA). NCM460 and HEK293T cells were maintained in DMEM medium (Gibco, Grand Island, NY, USA) supplemented with 10% fetal bovine serum (FBS, Gibco). LoVo cells were grown in F12K medium (Gibco) containing 10% FBS (Gibco). HCT116 cells were cultured in Iscov’s Modified Dulbecco’s Medium (IMDM) containing 10% FBS (Gibco). All cells were incubated in a humidified atmosphere containing 5% CO_2_ at 37°C.

### Reagents and cell transfection

Small interference RNAs (siRNAs) targeting BANCR (si-BANCR) and CSE1L (si-CSE1L), scramble control siRNA (si-Control), miR-203 mimic and its scramble control (miR-Control) were obtained from GenePharma Co. ltd (Suzhou, China). The cDNA sequences of CSE1L and BANCR were subcloned into pcDNA3.1 vector (Invitrogen, Carlsbad, CA, USA) to generate pcDNA-CSE1L and pcDNA-BANCR overexpression plasmids. All these oligomers or plasmids were transfected into HCT116 or LoVo cells cells by Lipofectamine 2000 reagent (Invitrogen) according to the protocols of manufacturer. Adriamycin (ADR) was purchased from Sigma-aldrich Co. ltd. (St. Louis, MO, USA).

### RT-qPCR assay

Total cellular RNA was extracted from tissues or cells using Trizol reagent (Invitrogen) and was reversely transcribed into cDNA first strand using M-MLV reverse transcriptase (Thermo Fisher Scientific, Rockford, IL, USA) and random primer (for BANCR, CSE1L, U6 snRNA and GAPDH) or specific reverse transcription (RT) primers for miR-203. Next, expression patterns of miR-203, U6 snRNA, BANCR, CSE1L and GAPDH were determined using quantitative primers and SYBR® Premix Ex Taq™ reagent (Takara). GAPDH was used to normalize expressions of BANCR and CSE1L, and U6 snRNA acted as endogenous control of miR-203. The RT primer for miR-203 was listed as follows: miR-203 (RT), 5’-GTCGTATCCAGTGCAGGGTCCGAGGTATTCGCACTGGATACGACCTAGTG-3’. Quantitative primers were presented as below: miR-203, 5’-GTGCAGGGTCCGAGGT-3’ (forward) and 5’-GCCGCGTGAAATGTTTAGG-3’ (reverse); U6 snRNA, 5’-CTCGCTTCGGCAGCACA-3’ (forward) and 5’-AACGCTTCACGAATTTGCGT-3’ (reverse); BANCR, 5’-ACAGGACTCCATGGCAAACG-3’ (forward) and 5’-ATGAAGAAAGCCTGGTGCAGT-3’ (reverse); CSE1L, 5’-CGCACCGTTTGTTGAGATTC-3’ (forward) and 5’-TGATGAGAGTAGGGATGTAGGG-3’ (reverse); GAPDH, 5’-GGGAGCCAAAAGGGTCAT-3’ (forward) and 5’-GAGTCCTTCCACGATACCAA-3’ (reverse).

### Western blot assay

Total proteins were extracted using RIPA buffer (Beyotime, Shanghai, China) containing protease inhibitor (cocktail, Roche, Basel, Switzerland) and then quantified by Pierce™ BCA Protein Assay Kit (Thermo Fisher Scientific). Next, 50 μg of each protein samples were separated by SDS-polyacrylamide gel electrophoresis (SDS-PAGE) and electrotransferred to nitrocellulose (NC) membranes (Millipore Corp. Bedford, MA, USA). Following blocked with 5% non-fat milk for 1 h at room temperature, the membranes were probed with primary antibodies against CSE1L (ab96755, 1:2000, Abcam, Cambridge, UK), and p53-binding protein (53BP1) (ab36823, 1:20000, Abcam), flap endonuclease 1 (FEN1) (AB109132, 1:5000, Abcam), β-actin (ab8227, 1:5000, Abcam) overnight at 4 °C. Then the membranes were further incubated with horseradish peroxidase conjugated goat-anti-rabbit secondary antibody (ab205178, 1:10000, Abcam) for 1 h at room temperature. At last, protein signals were detected using SuperSignal West Pico PLUS Chemiluminescent Substrate (Thermo Fisher Scientific).

### Luciferase assay

Partial sequences of BANCR and CSE1L 3’UTR containing putative miR-203 binding sites were amplified by PCR and constructed into pGL3-control vectors (Promega, Madison, WI, USA) to form wide type (WT)-BANCR and CSE1L 3’UTR-WT luciferase reporters. Moreover, GeneArt™ Site-Directed Mutagenesis System (Invitrogen) was used to generate mutant type (Mu)-BANCR and CSE1L 3’UTR-Mu luciferase reporters with mutant miR-203 binding sites. Then constructed reporters were co-transfected with pRL-TK vectors (Promega) and miR-203 mimic or miR-Control into LoVo and HCT116 cells. At 48 h post-transfection, luciferase activities were determined using dual-luciferase reporter assay system (Promega) referring to protocols of manufacturer.

### MTT assay

Cell proliferation patterns and cell survival rate under the treatment of different concentrations of ADR were measured using MTT assays. For the detection of cell proliferation patterns, MTT assay was performed at the time points (0, 24, 48, 72 h) after transfection. For the determination of cell survival rate, untransfected or transfected cells were treated with various concentrations of ADR (0, 20, 40, 80, 160, 320, 640, 1280 ng/ml) for 48 h, followed by the conduction of MTT assay. At the indicated time points after treatment, 20 μl of 5 mg/ml MTT solution (Sigma-Aldrich) was inoculated into 96-well plates for 4 h at 37°C. Then, medium was removed and 150 μl dimethyl sulfoxide (DMSO, Sigma-aldrich) was added into each well to dissolve formed formazan crystals. After shaking for 15 min, cell absorbance was measured at the wavelength of 490 nm.

### Cell apoptosis assay

Cell apoptosis were detected at 48 h posttransfection using eBioscience™ Annexin V-FITC Apoptosis Detection Kit (Invitrogen) following manufacturer’s instruction. Generally, collected LoVo and HCT116 cells were resuspended in 200 μl Binding Buffer (1×), and then stained with 5 μl Annexin V-FITC and 10 μl Propidium Iodide (PI, 20μg/ml) for 10 min at room temperature in the dark. Next, apoptotic rates were determined using a flow cytometry (FACScan; BD Biosciences, San Jose, CA, USA).

### Matrigel invasion assay

At 24 h after transfection, LoVo and HCT116 cells were collected and resuspended in medium containing 1% FBS. Then cells (1×10^5^) were plated into BD BioCoat BD matrigel invasion chambers precoated with matrigel (BD Biosciences). Medium containing 20% FBS were added into the lower chamber. At 24 h post incubation, cells on the upper surface of membranes were removed using a cotton swab, while cells invaded into the lower surface of the membranes were fixed using methanol for 30 min and stained with crystal violet solution (0.1%, Sigma-aldrich) for 20 min. Next, average cell numbers in 10 randomly selected fields were counted by microscope.

### Lentivirus production and infection

The shRNA sequences of BANCR were subcloned into pLKO.1 lentivirus vetor (Addgene, Cambridge, Massachusetts, USA) to obtain sh-BANCR lentivirus plasmid. pLKO.1 empty vector performed as sh-Control plasmid. Then, sh-Control or sh-BANCR lentivirus plasmid was transfected into HEK293T cells along with lentivirus packaging plasmids (psPAX2 and pMD2.G, Addgene). At 72 h upon transfection, cell supernatants containing sh-Control or sh-BANCR lentivirus were collected, respectively. Then HCT116 cells were infected with sh-Control or sh-BANCR lentivirus, followed by the screening of puromycin (Sigma-aldrich). After almost 7 days, stable lentivirus-transfected HCT116 cell lines were obtained.

### *In vivo* assay

All animal experiments were carried out following the national standard of the care and use of laboratory animals. Also, our study was approved by Institutional Committee for Animal Research. Five weeks old male Balb/c mice (n=24) were purchased from Hubei Research Center of Laboratory Animal (Wuhan, China). Next, these mice were randomly divided into 4 groups (sh-Control, sh-Control+ADR, sh-BANCR, sh-BANCR+ADR) with 6 mice in each group. HCT116 cells (5 × 10^6^) stably transfected with sh-Control or sh-BANCR were subcutaneously injected into the left flank of mice. At 6 days after injection, mice in sh-Control+ADR and sh-BANCR+ADR groups were administrated with ADR (1 mg/kg) every 3 days. Tumor volume was determined at the indicated time points (6, 9, 12, 15, 18, 21 days) after first injection. At 21 days upon cell inoculation, tumors were excised, imaged and weighted. Moreover, expressions of BANCR and CSE1L in xenograft tumors were determined by RT-qPCR and western blot assays, respectively.

### Statistical analysis

Data was gained from at least three independent experiments with the results presented as mean ± standard deviation (mean ± SD). One-way ANOVA or Student’s *t*-test was used to compare the difference between groups. Correlations between BANCR, CSE1L and miR-203 were analyzed by the Spearman’s test. *P* <0.05 meant that difference was statistically significant.

## Supplementary Material

Supplementary Figure
