# Supplementary material for: LncRNA BANCR promotes tumorigenesis and enhances adriamycin resistance in colorectal cancer
Source: Aging (Albany NY). 2018 Aug 22;10(8):2062–78. doi: 10.18632/aging.101530 (PMC6128424; doi:10.18632/aging.101530)
Supplement: Supplementary Figure [file aging-10-101530-s001.pdf]

SUPPLEMENTARY FIGURE

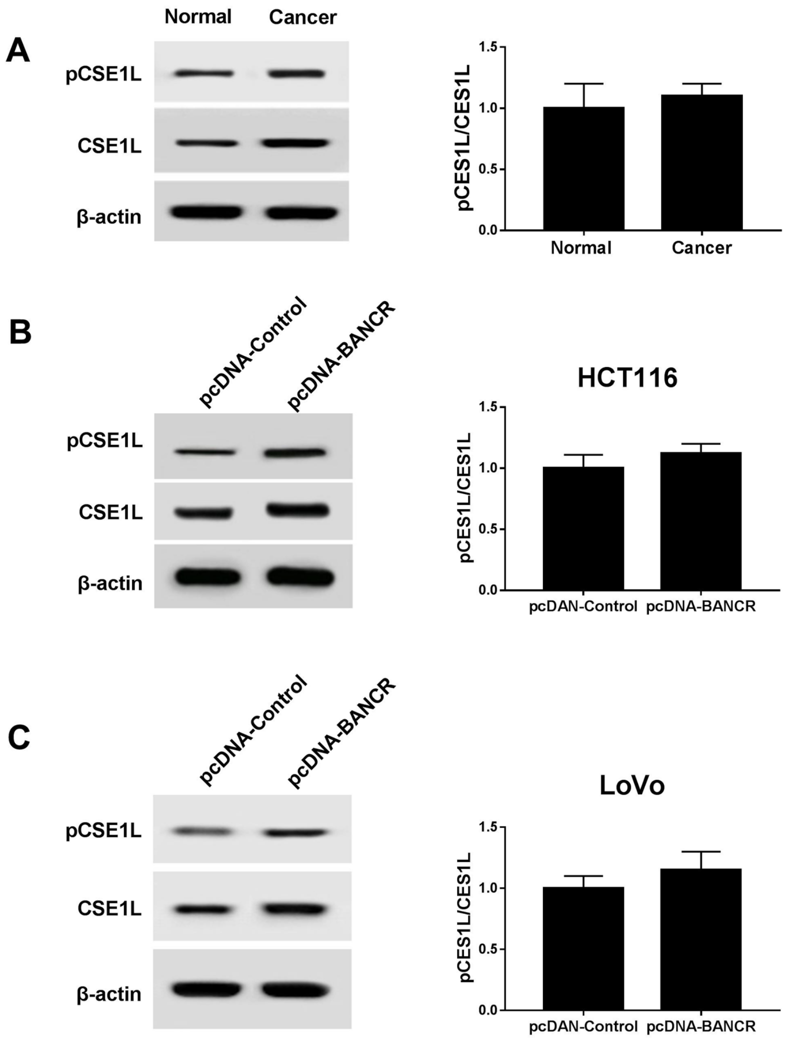

Figure S1. Phosphorylated CSE1L in CRC tissues and cells. (**A**) The ratio of pCSE1L/CSE1L was investigated in CRC tissues and normal control. (**B** and **C**) The effect of BANCR on pCSE1L/CSE1L ratio was evaluated in HCT116 and LoVo cells.
